# Pedigree and Functional Analysis of Two Cryptic *OTC* Variants Causing Ornithine Transcarbamylase Deficiency in Two Unrelated Chinese Male Patients

**DOI:** 10.1002/mgg3.70223

**Published:** 2026-04-28

**Authors:** Qingming Wang, Huimin Xiao, Fang Zhang, Hui Li, Juan Zhao, Haiming Yuan

**Affiliations:** ^1^ Dongguan Maternal and Child Health Care Hospital Dongguan China; ^2^ The Sixth Affiliated Hospital Guangdong Pharmaceutical University Dongguan China; ^3^ Huadu District People's Hospital of Guangzhou Guangzhou China

**Keywords:** inframe variant, minigene, *OTC*, OTCD, synonymous variant

## Abstract

**Background:**

Ornithine transcarbamylase deficiency (OTCD, MIM#311250) is a rare X‐linked urea cycle disorder causing hyperammonemia. While around 600 pathogenic *OTC* variants have been reported, cryptic changes like synonymous or in‐frame variants remain poorly characterized and are easily overlooked in routine screening.

**Methods:**

We analyzed the clinical and genetic profiles of two unrelated Chinese male patients clinically diagnosed with OTCD. Whole‐exome sequencing (WES) was performed to identify the genetic etiology. A minigene assay was then used to assess the splicing effect of the detected synonymous variant.

**Results:**

WES revealed two rare *OTC* variants: a synonymous variant (NM_000531.6: c.663G>A, p.Lys221Lys) and a novel *de novo* inframe variant (c.756_761dupAGCAGC, p.Ala253_Ala254dup). Minigene assay confirmed that the c.663G>A variant causes exon 6 skipping, leading to the deletion of 41 amino acids (c.541_663del, p.Glu181_Lys221del). Both variants were classified as clinically pathogenic, and the genotype–phenotype relationships were potentially established.

**Conclusion:**

Our study expands the mutation spectrum of *OTC* and emphasizes the importance of cryptic variants interpretation and the need for additional functional studies to verify the pathogenicity of these variants.

## Graphical abstract

Two unrelated Chinese male OTCD‐suspected patients harbored an *OTC* synonymous variant (c.663G>A, p.Lys221Lys) and a novel *de novo* in‐frame variant (c.756_761dupAGCAGC, p.Ala253_Ala254dup); minigene analysis confirmed the synonymous variant induces exon 6 skipping with 41 amino acid deletion (c.541_663del, p.Glu181_Lys221del).

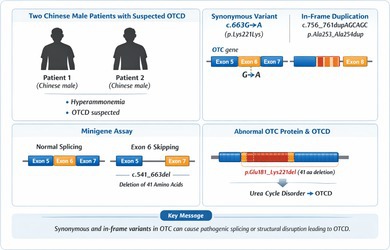

## 1 Introduction

Urea cycle disorders (UCDs) are a group of inherited metabolic conditions characterized by the triad of hyperammonemia, encephalopathy, and respiratory alkalosis, which result from deficiencies in enzymes or transporters involved in the urea cycle, impairing the conversion of ammonia into urea for excretion. The incidence of UCDs is estimated to be 1 in 35,000 live births, with high mortality (25%–50%) (Burgard et al. 2016; Nettesheim et al. 2017; Häberle et al. 2019; Simpson et al. updated 2025). Currently, at least 10 genes have been linked to UCDs, involving *NAGS*, *CPS1*, *ASS1*, *ASL*, *ARG1*, *SLC25A15*, *SLC25A13*, *OAT*, *CA5A*, and *OTC*, which exhibit an autosomal recessive or X‐linked pattern (Häberle et al. 2019).

Ornithine transcarbamylase deficiency (OTCD, MIM#311250), caused by a pathogenic variant in the *OTC* gene (MIM#300461), is the most common type of UCDs. OTCD is the only UCDs with an X‐linked inheritance pattern, and its estimated incidence ranges from 1 in 80,000 to 1 in 40,000 (Caldovic et al. 2015). OTCD is categorized into two clinical types according to the age of onset and severity: a severe neonatal‐onset disease in males (but rarely in females) and a post‐neonatal‐onset (also known as “late‐onset” or partial deficiency) disease in males and females. The timing of onset is indicative of the extent of enzyme activity deficiency (Posset et al. 2019). Currently, approximately 600 pathogenic variants in *OTC* have been reported in the literature. However, the molecular diagnosis still remains challenging for a few patients with clinical features suggestive of OTCD. It is partially attributed to some cryptic variants, such as synonymous or in‐frame variants, which are likely to be missed or classified as variant unknown significance (VUS) during variant interpretation.

In this study, we identified a synonymous variant (NM_000531.6: c.663G>A, p.Lys221Lys) and a novel *de novo* inframe insertion variant (c.756_761dupAGCAGC, p.Ala253_Ala254dup) in the *OTC* gene in two male patients from two unrelated Chinese families. Both patients exhibited characteristics typical for OTCD. Next, we conducted a systematic review of the clinical characteristics of OTCD patients with synonymous or inframe variants, with the primary aim of establishing a potential genotype–phenotype correlation.

## 2 Materials and Methods

### 2.1 Ethical Compliance

This study was approved by the Ethics Committee of Dongguan Maternal and Child Health Care Hospital (2022‐100). Written informed consent was obtained from the legal guardian for the publication of any potentially identifiable images or data in this manuscript.

### 2.2 Whole‐Exome Sequencing

Trio‐based whole‐exome sequencing was employed for the affected families to screen for causal variants. Genomic DNA was extracted from peripheral blood samples using QIAamp DNA Blood Mini Kit (Qiagen, Germany). Library preparation was operated using the Agilent SureSelect Human All Exon kit V5 (Agilent, Santa Clara, CA). The bcl2fastq2 Conversion Software (v2.20) was used for extracting Fastq files, and all reads were aligned to the human reference genome version (GRCh37/hg19) by using BWA (v0.2.10) with default parameters. The Genome Analysis Toolkit (GATK; v.3.7) HaplotypeCaller was performed for detecting variants. The aligned reads were visualized by using the Integrated Genome Viewer (IGV). Genomic variants were filtered based on their frequencies in the databases of the Genome Aggregation Database (https://gnomad.broadinstitute.org/) and in‐house database. Variants were analyzed based on previously reported variants in ClinVar and the Human Gene Mutation Database (HGMD). Candidate variants were validated using Sanger sequencing. The pathogenicity of the sequence variants was assessed according to ACMG/AMP guidelines (Richards et al. 2015).

### 2.3 In Vitro Minigene Assays

To verify the potential effects of the synonymous variant (c.663G>A, p.Lys221Lys) in *OTC*, an in vitro minigene splicing assay was performed. The *OTC* minigene construct included partial intron 5 (515 bp), full exon 6 (123 bp), and partial intron 6 (515 bp). Amplification was conducted using the following primer pair: forward 5′‐GGTAGGTACCCACCTGGCCAACTAACAGTA‐3′ and reverse 5′‐TTTCCTCGAGCATGTGGGGCTCAAATTTTC‐3′. The amplified products from both wild‐type and mutant sequences were cloned into the pcMINI vector. The recombinant vectors were then transiently transfected into HeLa and HEK293T cell lines. After 48 h, total RNA was extracted from the transfected cells using TRIzol reagent (9109; TaKaRa, Japan). For RT‐PCR analysis, a specific primer pair: forward 5′‐CTAGAGAACCCACTGCTTAC‐3′ and reverse 5′‐TAGAAGGCACAGTCGAGG‐3′ was used to amplify the cDNA. Finally, PCR products were verified by Sanger sequencing and visualized with electrophoresis on a 1.2% agarose gel.

## 3 Results

### 3.1 Patient 1

The proband was a 4‐year‐4‐month‐old male patient with a healthy nonconsanguineous couple. He was delivered at 38 weeks gestational age after an uneventful pregnancy, with a normal birth measurement: weight 3.0 kg, length 50 cm and head circumference 34 cm. His 7‐year‐old brother was totally healthy. At the age of 1 year 2 months, the male patient became irritable after eating. He developed intermittent limb twitching lasting 2–4 min each time, with an average of six episodes every day. It was followed by non‐projectile vomiting (2–7 episodes/day) and progressive lethargy. On this occasion, laboratory tests showed obvious hyperammonemia (229.4 μmol/L; normal: 18–72 μmol/L). The urine sample showed significant elevation of orotic acid (20 μmol/mmol creatinine; normal: < 10 μmol/mmol creatinine). Plasma amino acid analysis revealed a normal level of citrulline (12 μmol/L; normal: 10–50 μmol/L) and a low level of arginine (35 μmol/L; normal: 40–120 μmol/L). Liver function tests revealed increased indirect bilirubin (5.2 μmol/L; normal: 0–5 μmol/L), aspartate aminotransferase (41.0 U/L; normal: 0–38 U/L), alanine aminotransferase (72.1 U/L; normal: 0–50 U/L), and total bile acid (25.3 μmol/L; normal: 0–10 μmol/L). Since then, the patient has been treated with arginine supplements (100 mg/kg/day) and a low‐protein diet (2 g/kg/day). He has remained generally well, although he occasionally suffers from episodes of hyperammonemia, particularly precipitated by stressors such as fasting, physical or psychological trauma.

**FIGURE 1:**
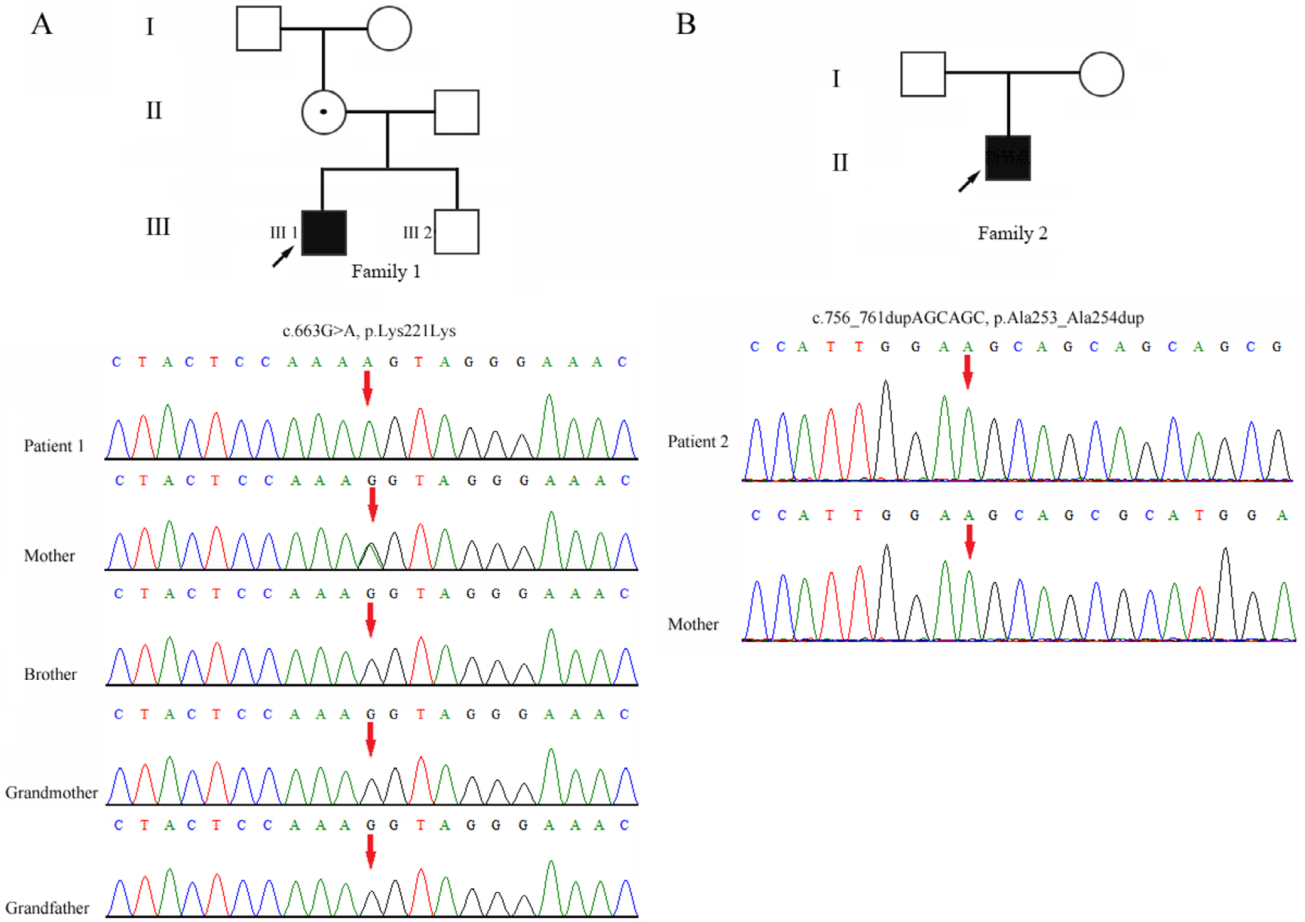
Pedigree of two families and genetic testing results. Pedigree of the family 1 with segregation of the identified *OTC* variant. A hemizygous synonymous variant in *OTC* (c.663G>A, p.Lys221Lys) was detected in patient 1 (III‐1), which he inherited from the asymptomatic mother. His healthy brother was wildtype (III‐2). The variant was absent from the grandfather and grandmother. Pedigree of the family 2. A *de novo* hemizygous inframe variant in *OTC* (c.756_761dupAGCAGC, p.Ala253_Ala254dup) was detected in patient 2. Square: Male; Round: Female; Arrow: Proband; Filled: Affected individuals; Black dot: Carrier.

**FIGURE 2:**
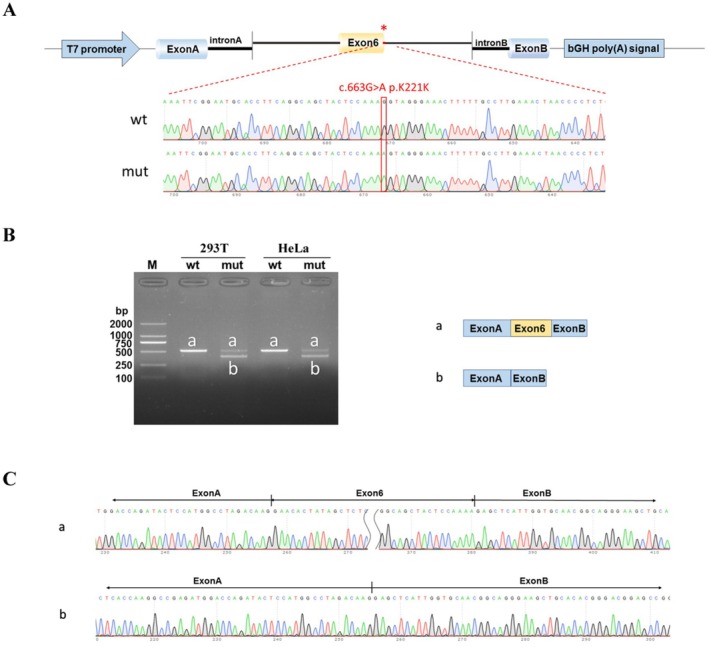
Minigene assay for *OTC* c.663G>A variant and schematic diagram of the splicing pattern. (A) The construction of OTC‐pcMINI minigene plasmid; (B) Gel electrophoresis of RT‐PCR revealed a single band for wild‐type (wt) and two bands for mutant‐type (mut); (C) minigene product sequencing demonstrated that the wild‐type minigene formed normal mRNA, but the c.663G>A variant in *OTC* caused a splicing abnormality, which abrogated the canonical splice site of intron 6, resulting in exon 6 skipping.

WES identified a hemizygous synonymous variant in the *OTC* gene (c.663G>A, p.Lys221Lys), which was inherited from an asymptomatic mother. The variant was not detected in maternal grandparents, showing a *de novo* event (PS2). This variant was also not detected in the proband's healthy brother (Figure 1A) and had been previously reported in late‐onset OTCD male patients in two independent studies (Shimadzu et al. 1998; Fujisawa et al. 2015; PP1_moderate). The c.663G>A variant was absent from population databases including the Genome Aggregation Database (gnomAD) and the 1000 Genomes Project (PM2_supporting). Next, minigene assays were performed and confirmed that the variant disrupted the 3′ splice acceptor site of exon 6 and caused exon 6 skipping. The final annotation was an in‐frame deletion variant: c.541_663del, p.Glu181_Lys221del (PM4) (Figure 2). Furthermore, the patient's clinical presentation was strikingly similar to OTCD (PP4). Based on these findings, the variant was classified as pathogenic according to the ACMG/AMP guidelines (PS2 + PM2_supporting+PM4 + PP1_moderate+PP4) (PS, pathogenic strong; PM, pathogenic moderate; PP, pathogenic supporting).

### 3.2 Patient 2

Patient 2 was a male neonate born to nonconsanguineous parents. The pregnancy and delivery were uneventful. He had normal birth measurement but experienced seizures, vomiting, and coma on the third day of life. Laboratory tests showed obvious hyperammonemia (1046 μmol/L; normal: 18–72 μmol/L). The urine sample showed a markedly abnormal increase of orotic acid excretion (33 μmol/mmol creatinine; normal: < 10 μmol/mmol creatinine). Plasma amino acid analysis revealed low levels of citrulline (4.6 μmol/L; normal: 10–50 μmol/L) and arginine (24.5 μmol/L; normal: 40–120 μmol/L). Liver function tests showed markedly increased direct bilirubin (17.2 μmol/L; normal: 0–6.8 μmol/L), glutamate ester transaminase (112 U/L; normal: 0–38 U/L), and gamma‐glutamyl transpeptidase (158 U/L; normal: 11–50 U/L). Eventually, the patient died on the fifth day due to the severe hyperammonemia and respiratory alkalosis.

WES identified a novel *de novo* hemizygous inframe insertion variant (c.756_761dupAGCAGC, p.Ala253_Ala254dup) in the *OTC* gene (Figure 1B) (PS2). The variant was absent from public population databases, including gnomAD and the 1000 Genomes Project (PM2_supporting). The clinical phenotype of the patient was highly consistent with OTCD (PP4). Next, protein modeling of the mutant (c.756_761dup/p.Ala253_Ala254dup) was performed using AlphaFold3 (https://golgi.sandbox.google.com/) to assess its impact on the 3D structure, with subsequent analysis in PyMOL. It showed that the variant caused a conformational rearrangement in the local peptide structure from residue 253, resulting in altered electrostatic potential and hydrophobic distribution. Such changes were likely to impact protein function by modulating catalytic activity, substrate binding, stability and interaction networks (PP3) (Supporting Information). Based on these lines of evidence, the variant was classified as likely pathogenic according to the ACMG/AMP guidelines (PS2 + PM2_supporting + PP3 + PP4) (PS, pathogenic strong; PM, pathogenic moderate; PP, pathogenic supporting).

## 4 Discussion

Pathogenic variants in *OTC* have been linked to OTCD through a haploinsufficiency mechanism. The *OTC* gene, located at Xp11.4, spans a total length of 73 kb, contains 10 exons, and encodes a protein of 354 amino acids (Horwich et al. 1984). It is predominantly expressed in the liver, with lower expression levels in intestinal mucosal cells (Caldovic et al. 2015). The OTC enzyme catalyzes the mitochondrial reaction between ornithine and carbamoyl phosphate to form citrulline, which is subsequently transported to the cytoplasm to continue participating in the urea cycle (Morris Jr. 2002).

**FIGURE 3:**
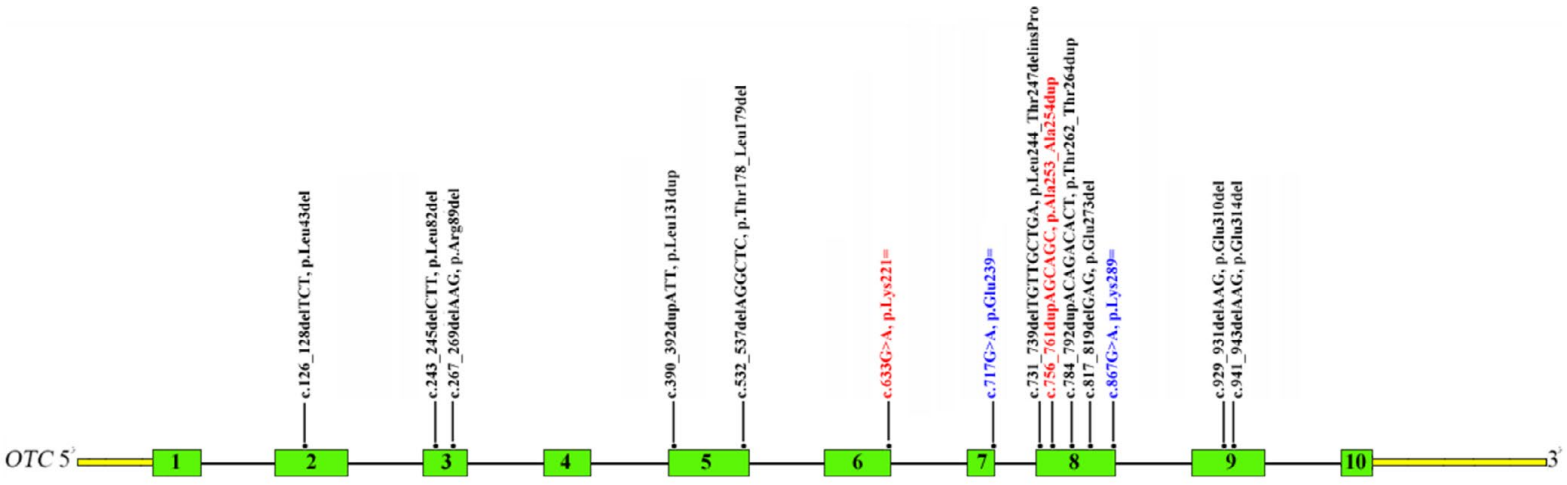
Schematic representation of *OTC* inframe and synonymous variants identified to date. The structure of *OTC* contains 10 exons (green rectangles), introns (black horizontal line), and the untranslated region (yellow rectangle). Black: Variants identified in the literature; Blue: Synonymous variants; Red: Variants detected in this study.

Currently, approximately 600 pathogenic variants in *OTC* have been recorded in the HGMD. *OTC* missense variants and null variants (nonsense, frameshift, splicing) are frequently detected in OTCD patients, accounting for 62% and 36%, respectively. However, the molecular diagnosis still remains challenging for a few OTCD patients due to poor understanding of cryptic variants detected. Currently, only three synonymous variants (c.663G>A, c.717G>A, c.867G>A) have been reported in the literature (Shimadzu et al. 1998; Fujisawa et al. 2015; Tuchman et al. 1997; Clarkston et al. 2021; Storkanova et al. 2013; Table 1, Figure 3). However, no functional experiments are performed to verify their pathogenicity. In this study, we identified a previously reported synonymous variant (c.663G>A, p.Lys221Lys) in *OTC* in a Chinese male patient with clinical features suggestive of OTCD. In order to seek the convincing evidence, in vitro minigene assay was performed and showed that the variant disrupted the 3′ splice acceptor site of exon 6, resulting in exon 6 skipping. Eventually, the variant was annotated as an in‐frame deletion variant (c.541_663del, p.Glu181_Lys221del), which was evaluated as clinically pathogenic according to the ACMG/AMP guidelines. Thus, the patient was clinically and molecularly diagnosed with OTCD. Next, we systematically analyzed the clinical phenotypes of seven individuals with synonymous variants in *OTC*, including our patient 1 (Table 1). Among these patients, the peak values of blood ammonia exhibited significant variability. It was observed that individuals harboring synonymous variants often presented with a late‐onset phenotype. Certainly, it was required to collect more individuals with *OTC* synonymous variants to further confirm the findings.

**TABLE 1:** Overview of synonymous variants and phenotypes observed in OTCD patients.

Number	Variant	Exon	Gender	Age of onset	Type	Nature of mutation	The peak of plasma ammonia (μmol/L)	References
1	c.663G>A,p.Lys221Lys	6	Male	1.2 years	LO	Inherited	229.4	This study
2	c.663G>A,p.Lys221Lys	6	Male	4 years	LO	*De novo*	NA	Shimadzu et al. (1998)
3	c.663G>A,p.Lys221Lys	6	Male	7 years	LO	Inherited	178	Fujisawa et al. (2015)
4	c.663G>A,p.Lys221Lys	6	Male	4 years	LO	Inherited	> 1983	Fujisawa et al. (2015)
5	c.717G>A,p.Glu239Glu	7	Female	NA	NA	NA	NA	Tuchman et al. (1997)
6	c.717G>A,p.Glu239Glu	7	Female	1.3 years	LO	NA	447	Clarkston et al. (2021)
7	c.867G>A,p.Lys289Lys	8	Male	1 year	LO	*De novo*	4500	Storkanova et al. (2013)

Abbreviations: LO, late‐onset; NA, not available.

In this study, we also identified a novel de novo inframe insertion variant (c.756_761dupAGCAGC, p.Ala253_Ala254dup) in *OTC* in our patient 2. The male neonate presented with hyperammonemia, vomiting, coma, respiratory alkalosis, low citrulline and arginine concentration, and elevated orotic acid excretion, which pointed to OTCD. Currently, the pathogenic mechanism of OTCD caused by inframe variants in *OTC* remains elusive (Tuchman et al. 2002; Makris et al. 2021). The three‐dimensional structure of the OTC protein predicted that the variant had a deleterious effect on the protein structure. This variant was classified as clinically likely pathogenic according to ACMG/AMP guideline.

Next, we conducted the genotype–phenotype analysis for all patients with *OTC* inframe variants. Most reported inframe variants were located in exons 8 and 9 (Figure 3). Currently, a total of 8 inframe deletion variants involving 14 individuals have been reported, and the majority were diagnosed with late‐onset OTCD (Table 2). The average age of onset was 4.55 years, with an average peak blood ammonia level of 284 μmol/L (Lu et al. 2020; Storkanova et al. 2013; Tuchman and Plante 1995; Makris et al. 2021; Shimadzu et al. 1998; Calvas et al. 1998; Ségues et al. 1996; Ducich et al. 2020; Toquet et al. 2021; Tuchman et al. 1994; Yamaguchi et al. 2006; Table 2). However, three individuals carrying inframe insertion variants in *OTC* displayed early‐onset OTCD (Tuchman et al. 2002; Makris et al. 2021; Table 2). Thus, it was speculated that inframe deletion variants may be associated with late‐onset OTCD, whereas inframe insertion variants may lead to early‐onset OTCD, which needed to be further investigated due to small sample sizes.

**TABLE 2:** Overview of inframe variants and phenotypes observed in OTCD patients.

Number	Variant	Exon	Gender	Age of onset	Type	Nature of mutation	The peak of plasma ammonia (μmol/L)	References
1	c.126_128delTCT, p.Leu43del	2	Female	1.1 years	LO	*De novo*	300	Lu et al. (2020)
2	c.126_128delTCT, p.Leu43del	2	Male	Neonatal	EO	*De novo*	5000	Storkanova et al. (2013)
3	c.243_245delCTT, p.Leu82del	3	Female	NA	NA	NA	NA	Tuchman and Plante (1995)
4	c.267_269delAAG, p.Arg89del	3	Female	NA	NA	NA	NA	Makris et al. (2021)
5	c.532_537delACGCTC, p.Thr178_Leu179del	5	Male	6 days	EO	NA	NA	Shimadzu et al. (1998)
6	c.731_739delTGTTGCTGA, p.Leu244_Thr247delinsPro	8	Female	5 years	LO	NA	NA	Calvas et al. (1998)
7	c.817_819delGAG, p.Glu273del	8	Male	0.8 year	LO	Inherit	NA	Ségues et al. (1996)
8	c.817_819delGAG, p.Glu273del	8	Male	7 years	LO	Inherit	363	Ducich et al. (2020)
9	c.817_819delGAG, p.Glu273del	8	Male	18 years	LO	NA	272	Toquet et al. (2021)
10	c.929_931delAAG, p.Glu310del	9	Male	2 years	LO	NA	200	Tuchman et al. (1994)
11	c.929_931delAAG, p.Glu310del	9	Male	2 years	LO	NA	200	Tuchman et al. (1994)
12	c.929_931delAAG, p.Glu310del	9	Male	2.1 years	LO	*De novo*	257	Lu et al. (2020)
13	c.929_931delAAG, p.Glu310del	9	Male	6 months	LO	Inherit	396	Storkanova et al. (2013)
14	c.941_943delAAG, p.Glu314del	9	Female	NA	NA	NA	NA	Yamaguchi et al. (2006)
15	c.390_392dupATT, p.Leu131dup	5	Female	NA	EO	NA	NA	Tuchman et al. (2002)
16	c.784_792dupACAGACACT, p.Thr262_Thr264dup	8	Male	NA	NA	NA	NA	Makris et al. (2021)
17	c.756_761dupAGCAGC, p.Ala253_Ala254dup	8	Male	Neonatal	EO	*De novo*	1046	This study

Abbreviations: EO, early‐onset; LO, late‐onset; NA, not available.

## 5 Conclusion

In conclusion, we identified a synonymous variant (c.663G>A, p.Lys221Lys) and a novel inframe variant (c.756_761dup, p.Ala253_Ala254dup) in *OTC* in two unrelated Chinese male patients diagnosed with OTCD. Our findings expanded the *OTC* mutation spectrum and highlighted that cryptic variants may be a potential cause of disease, which need additional functional studies to verify the pathogenicity. Furthermore, we established the potential genotype–phenotype relationship for synonymous and inframe variants, which need to be further studied.

## Author Contributions

Q.W. drafted the first versions of the manuscript. H.Y. was responsible for the design of the project, data analysis, and revised the manuscript. H.X. made the clinical evaluation. F.Z. and J.Z. performed the minigene study. H.L. performed molecular modeling. J.Z. provided financial support. All authors read and approved the final manuscript.

## Funding

This study was financed by the Huadu District Basic and Applied Basic Research Joint Funding Project (District‐Hospital Collaboration), Guangzhou (Grant No. 23HDQYLH15). The funding body participated in the design, experimental operation, and result interpretation of the project.

## Ethics Statement

This study was approved by the Ethics Committee of Dongguan Maternal and Child Health Care Hospital. Written informed consent was obtained from the legal guardians for the publication of any potentially identifiable images or data included in this article.

## Conflicts of Interest

The authors declare no conflicts of interest.

## Supporting information


**Figure S1:** The impact of the mutation on the tertiary structure of OTC protein. Note: The duplicated amino acid structure is highlighted in magenta, the yellow dash lines represent the H‐band.
**Figure S2:** The impact of the mutation on electrostatic potential of OTC protein. Note: the default color scheme is red for negative potential, white at zero, and blue for positive.
**Figure S3:** Effect of mutation on protein hydrophobicity. Note: The default coloring is from dark cyan for most hydrophilic through white to dark goldenrod for most hydrophobic.

## Data Availability

The datasets used and analyzed during the current study are available from the corresponding author on reasonable request.
