# Supplementary material for: Pedigree and Functional Analysis of Two Cryptic OTC Variants Causing Ornithine Transcarbamylase Deficiency in Two Unrelated Chinese Male Patients
Source: Mol Genet Genomic Med. 2026 Apr 28;14(5):e70223. doi: 10.1002/mgg3.70223 (PMC13125405; doi:10.1002/mgg3.70223)
Supplement: Supplementary file 1 — Figure S1: The impact of the mutation on the tertiary structure of OTC protein. Note: The duplicated amino acid structure is highlighted in magenta, the yellow dash lines represent the H‐band. Figure S2: The impact of the mutation on electrostatic potential of OTC protein. Note: the default color scheme is red for negative potential, white at zero, and blue for positive. Figure S3: Effect of mutation on protein hydrophobicity. Note: The default coloring is from dark cyan for most hydrophilic through white to dark goldenrod for most hydrophobic. [file MGG3-14-e70223-s001.docx]

**Pedigree and functional analysis of two** **cryptic *OTC* variants causing ornithine transcarbamylase deficiency in two unrelated Chinese male patients**

Qingming Wang^1^, Huimin Xiao^2^, Fang Zhang^1^, Hui Li^3^, Juan Zhao^3#^, Haiming Yuan^1^^#^

1. Dongguan Maternal and Child Health Care Hospital. Dongguan 523120, China

2. The sixth affiliated hospital guangdong pharmaceutical university. Dongguan 523953, China

3. Huadu District People’s Hospital of Guangzhou. Guangzhou 510800, China

Qingming Wang (Associate Professor, PhD) Email:[wqm0404@sina.com](mailto:haimingyuan@sina.cn)

ORCID: 0009-0009-7053-305X

Huimin Xiao (Associate Professor, MM) Email:drxhm8212@163.com

Fang Zhang (Assistant Professor, MM) Email:1548895223@qq.com

Hui Li (Assistant Professor, MM) Email:31542528@qq.com

^#^Correspondence

Juan Zhao (Professor, PhD) Email: [lulu1984zhao@aliyun.com](mailto:lulu1984zhao@aliyun.com)

Haiming Yuan (Professor, PhD) Email: [haimingyuan@sina.cn](mailto:haimingyuan@sina.cn) ORCID: 0000-0002-8510-0419

**Method**

**Molecular modeling**

The physicochemical properties of both the wild-type and mutant OTC proteins were analyzed using the ProtParam tool available on the ExPASy (https://web.expasy.org/protparam/). Additionally, the secondary structure predictions for the wild-type and mutant OTC proteins were performed using the SOPMA program (https://web.expasy.org/protparam/). The AlphaFold3 tool (https://golgi.sandbox.google.com/) was employed to establish the protein crystal structures and analyze the mutant sites of OTC. Then, the Pymol software was used to illustrate the molecular structures of the wild-type and mutant forms of the target genes. ChimeraX visualization software (https://www.cgl.ucsf.edu/chimerax/) was employed to generate electrostatic potential maps and analyze the changes in surface electrostatic potential before and after the mutation. The potential for structural disruption caused by the mutation was predicted using the Missense3D (http://missense3d.bc.ic.ac.uk/~missense3d/).

**Result**

The physicochemical properties of the OTC protein before and after the mutation were almost no difference. SOPMA showed a secondary structure change with the insertion of resides 253 and 254. The two extra amino acids residues extend the peptide backbone architecture (Fig.S1), causing a conformational rearrangement in the local spatial structure of the peptide from residue 253. Multiple new hydrogen bonds are observed post-mutation (Fig.S2), primarily involving the newly inserted Ala255. And the electrostatic potential in ChimeraX revealed that the mutation leads to a redistribution of the local surface charge. In the wild-type structure, this region is predominantly characterized by a significant negative electrostatic potential (Fig.S2). However, post-mutation, the electrostatic potential in this area is markedly weakened, shifting towards neutrality (increase in white areas). The hydrophobic distribution of the protein showed reduced hydrophobicity near the mutation site, with a significant increase in hydrophilicity (Fig.S3). Such conformational change may lead to alterations in the catalytic activity or substrate binding capacity, protein stability and interactions with other domains or subunits. Therefore, this mutation can impair the normal function of OTC protein.


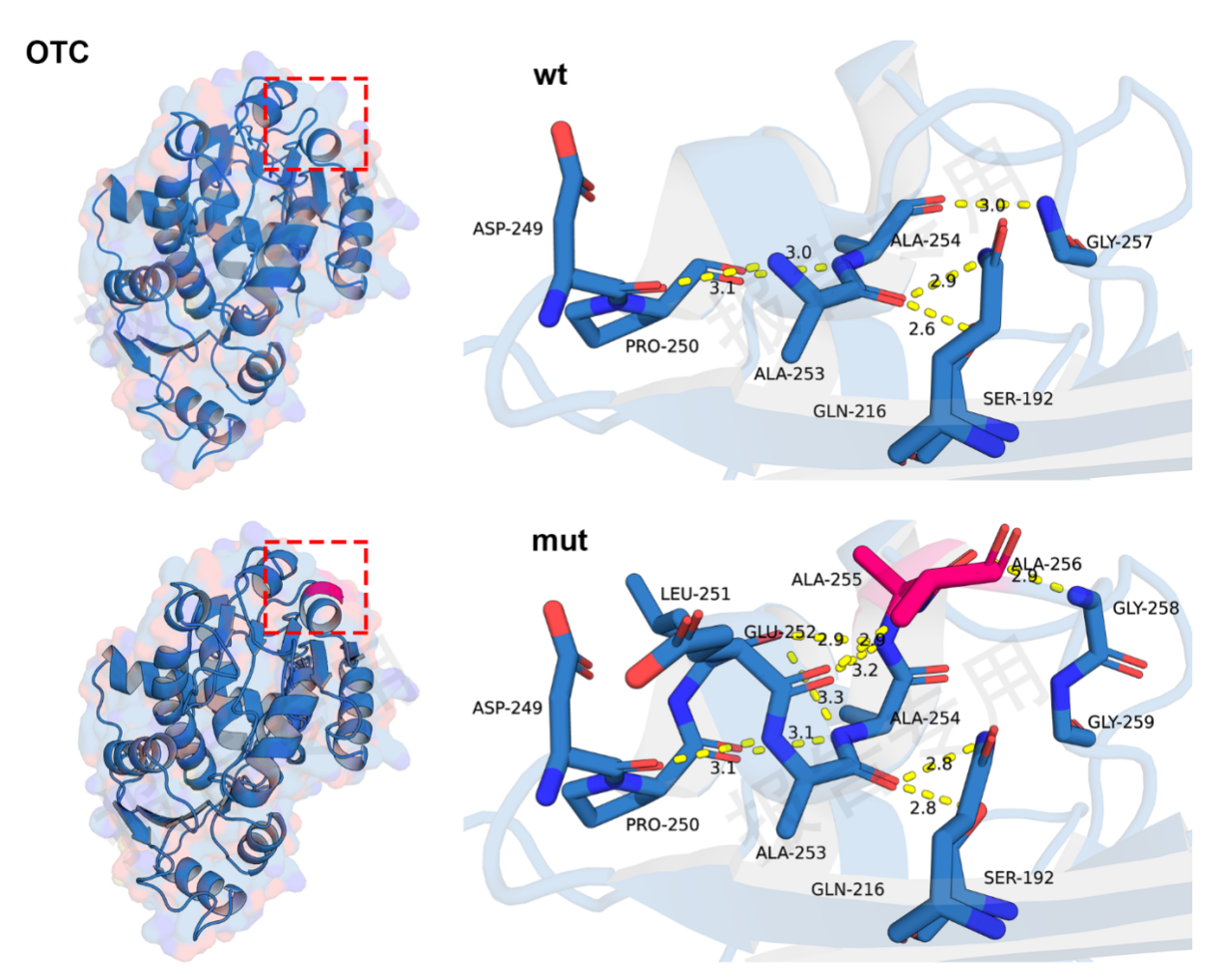


Fig. S1 The impact of the mutation on the tertiary structure of OTC protein

Note: The duplicated amino acid structure is highlighted in magenta, the yellow dash lines represent the H-band.


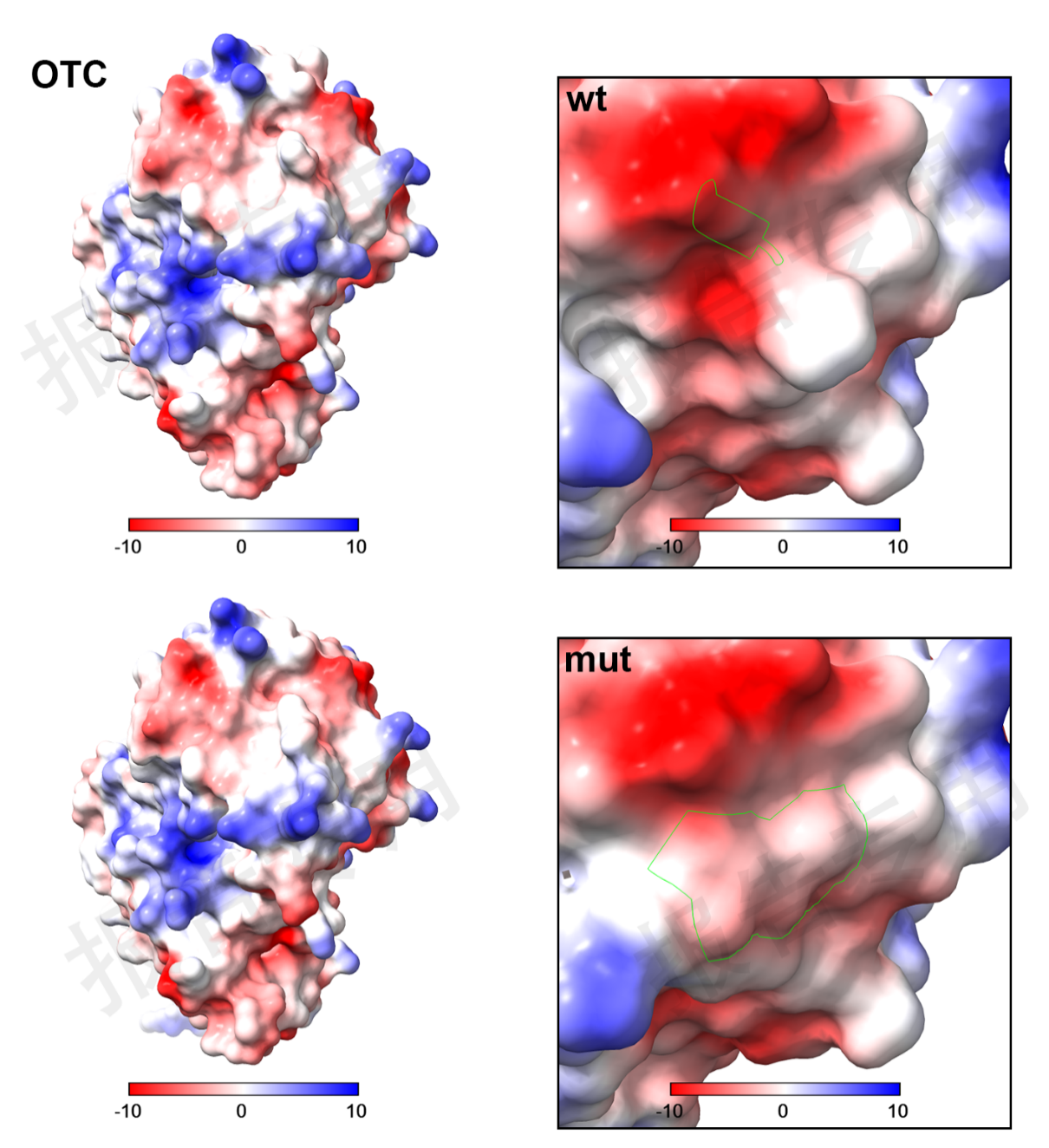


Fig. S2 The impact of the mutation on electrostatic potential of OTC protein

Note: the default color scheme is red for negative potential, white at zero, and blue for positive.


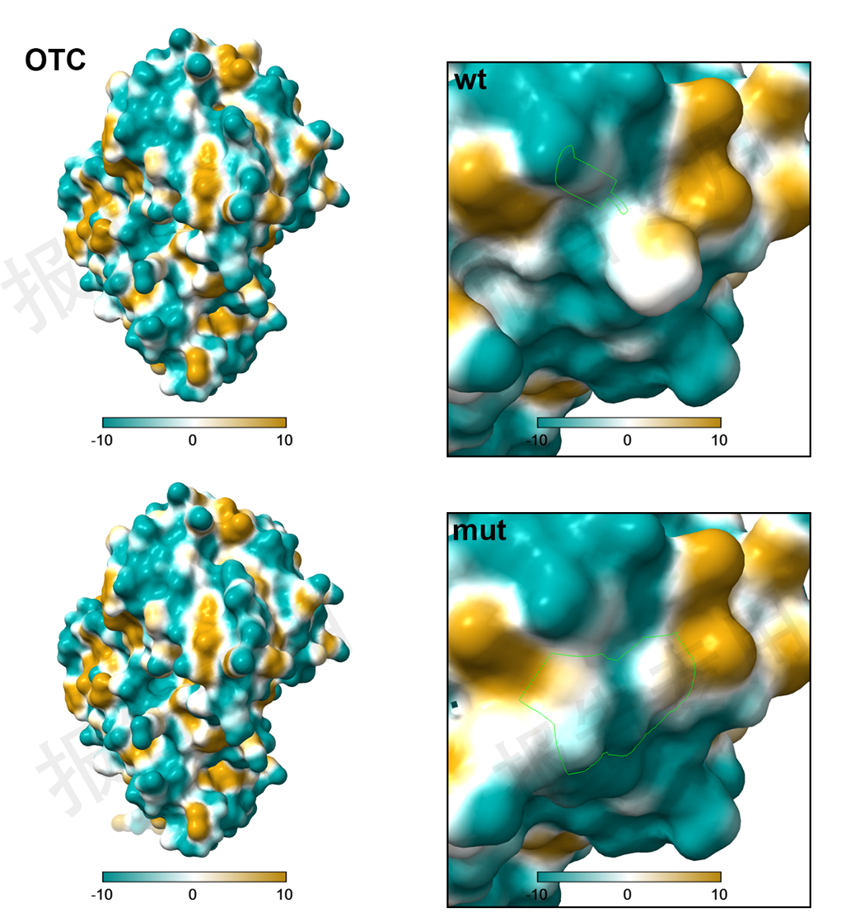


Fig. S3 Effect of mutation on protein hydrophobicity

Note: The default coloring is from dark cyan for most hydrophilic through white to dark goldenrod for most hydrophobic.
